# Targeting the MDM2-p53 Interaction with Siremadlin: A Promising Therapeutic Strategy for Treating *TP53* Wild-Type Chronic Lymphocytic Leukemia

**DOI:** 10.3390/cancers17020274

**Published:** 2025-01-16

**Authors:** Erhan Aptullahoglu, Mohammed Howladar, Jonathan P. Wallis, Helen Marr, Scott Marshall, Julie Irving, Elaine Willmore, John Lunec

**Affiliations:** 1 Biosciences Institute & Newcastle University Cancer Centre, Medical Faculty, Newcastle University, Newcastle upon Tyne NE2 4HH, UK; erhan.aptullahoglu@bilecik.edu.tr (E.A.); m.howladar2@newcastle.ac.uk (M.H.); julie.irving@ncl.ac.uk (J.I.); elaine.willmore@ncl.ac.uk (E.W.); 2 Department of Molecular Biology and Genetics, Faculty of Science, Bilecik Şeyh Edebali University, 11100 Bilecik, Türkiye; 3 Department of Haematology, Freeman Hospital, Newcastle upon Tyne NHS Foundation Trust, Newcastle upon Tyne NE7 7DN, UK; 4 Department of Haematology, City Hospitals Sunderland NHS Trust, Sunderland SR4 7TP, UK

**Keywords:** MDM2-p53 antagonists, siremadlin (HDM201), chronic lymphocytic leukemia (CLL), p53, p53-dependent apoptosis

## Abstract

**Background:** Chronic lymphocytic leukemia (CLL) treatment has transitioned from traditional chemotherapy to more targeted therapies, but challenges such as resistance and suboptimal responses persist. This study aimed to evaluate HDM201, a second-generation MDM2-p53 binding antagonist, as a novel therapeutic strategy for CLL, with a focus on its effectiveness across different *TP53* genetic contexts. **Methods:** We utilized a panel of B cell leukemia-derived cell lines with varying *TP53* statuses, including *TP53*-knockout (KO) derivatives of the human B cell line Nalm-6, and assessed the impact of HDM201 on primary CLL samples with both *TP53* wild-type and mutant backgrounds. **Results:** Our results revealed that *TP53* wild-type and heterozygous *TP53*-KO Nalm-6 cells were sensitive to HDM201, whereas homozygous *TP53*-KO cells and B cells with *TP53* mutations exhibited significant resistance. Resistance was also noted in primary CLL samples with *TP53* mutations. HDM201 effectively stabilized p53 and induced apoptosis in *TP53* wild-type cells but had limited efficacy in *TP53* mutant cells. **Conclusions:** These findings indicate that HDM201 holds promise as an additional targeted therapy option for wild-type *TP53* CLL. The results underline the importance of *TP53* status in predicting treatment efficacy and highlight the potential of HDM201 as a valuable addition to explore in CLL therapy. Future research should focus on identifying additional biomarkers of response and exploring the optimal way to include HDM201 in combination therapies to improve treatment outcomes in CLL.

## Simple Summary

This study explores HDM201, a second-generation MDM2-p53 binding antagonist, as a potential novel treatment for chronic lymphocytic leukemia (CLL). Using a range of B cell lines and primary CLL samples with different *TP53* statuses, we found that HDM201 effectively targets *TP53* wild-type and heterozygous *TP53*-KO cells but shows limited efficacy in *TP53* mutant and homozygous *TP53*-KO cells. HDM201 stabilizes p53 and induces apoptosis in sensitive cells, demonstrating its potential as a targeted therapy for *TP53* wild-type CLL cases. This study highlights the importance of *TP53* status in predicting treatment response and suggests further research on resistance mechanisms and combination therapies.

## 1. Introduction

Chronic lymphocytic leukemia (CLL) is one of the most common types of leukemia in adults [1], characterized by the accumulation of dysfunctional B lymphocytes in the blood, bone marrow, and lymphoid tissues [2,3]. Over the past 25 years, significant advancements have reshaped our understanding and management of CLL. Landmark studies published in 1999 [4,5] highlighted the critical role of somatic hypermutation of the *IGHV* genes in prognosis, driving extensive research into the biology, genetic factors, and diagnostic technologies relevant to the clinical management of CLL. These advances have not only propelled research within CLL but have also influenced the broader field of B cell malignancies. Despite significant progress, challenges remain due to the unique biological and clinical features of CLL.

Recent decades have witnessed a paradigm shift in CLL treatment, moving from traditional purine analog-based chemotherapy to novel therapeutic approaches, including the use of chemo-immunotherapy and B cell receptor (BCR) antagonists [6,7,8]. While chemo-immunotherapy historically provided durable disease control, its limitations in managing relapsed and high-risk cases [9,10,11] have highlighted the need for new strategies. The introduction of targeted therapies, including Bruton’s tyrosine kinase (BTK) inhibitors and BCL-2 (B cell lymphoma 2) inhibitors, has markedly improved treatment efficacy and tolerability, particularly for older patients with complex conditions. These therapies have largely replaced older treatments in both frontline and relapsed settings. Looking ahead, novel approaches such as bi-specific antibody therapies [12,13] and third-generation BTK inhibitors hold promise for tackling difficult cases like Richter’s transformation and heavily pre-treated CLL [14,15]. Nevertheless, in CLL patients, the predominant resistance mechanism to the BTK inhibitor ibrutinib is mutations in the *BTK* gene [16,17,18], and similar resistance mechanisms are also observed with next-generation inhibitors such as acalabrutinib [19]. There is, therefore, a need for novel therapeutic strategies to overcome these resistance challenges, improve treatment efficacy, and address a broader range of patient needs.

Given the crucial role of p53 in preventing abnormal cell proliferation and maintaining genomic integrity, there is a strong interest in developing non-genotoxic pharmacological strategies to modulate p53, aiming to enhance the selectivity and effectiveness of cancer cell elimination [20,21]. Importantly, compared to other cancers, *TP53* deletions and/or mutations are relatively infrequent in CLL at diagnosis, occurring in approximately only 10% of cases [22,23]. It is worth noting that, more generally, approximately 50% of human cancers harbor mutations in the *TP53* gene [24,25,26]. Furthermore, over 17% of tumors exhibit amplification and overexpression of the *MDM2* gene, which encodes the primary negative regulator of p53. These alterations, whether occurring individually or together, are associated with poor prognosis and treatment resistance [27,28,29]. Emerging therapies targeting the non-genotoxic activation of functional p53 protein offer promising avenues for enhancing treatment outcomes in CLL. The activity and levels of p53 are primarily regulated by its interaction with MDM2, a human homolog of the murine double-minute 2 protein [30]. MDM2 acts as an E3 ubiquitin ligase, controlling p53’s stability through ubiquitin-dependent proteasomal degradation [30]. Cellular stress induces the post-translational modifications of p53, resulting in disruption of the p53-MDM2 interaction, resulting in the release and accumulation of p53 to activate its target genes involved in cell cycle arrest, apoptosis, or senescence [30,31]. Nutlin-3 was the first selective MDM2-p53 binding antagonist (MDM2 inhibitor) shown to activate p53 and downstream signaling in preclinical models. Several new-generation MDM2 inhibitors have been developed, including RG7388 [32], HDM201 [33], AMG232 [34], DS-3032 [35], ASTX295 [36], and MI-77301 [37], which have been shown to reduce cancer cell viability and proliferation in preclinical models, including leukemia and lymphoma cells [38,39,40,41,42,43,44]. Most of these compounds are also currently being evaluated in clinical trials, both as monotherapies [45,46] and in combination therapies [47,48,49,50]. These non-genotoxic compounds bind specifically to the p53-binding pocket of MDM2 [51], stabilizing p53 and activating the p53 pathway.

A number of second-generation MDM2 inhibitors, including Siremadlin (HDM201), have progressed to evaluation in several recent and ongoing clinical trials for their efficacy in treating cancers with wild-type p53. Results from a first-in-human phase I study of HDM201 in patients with advanced wild-type *TP53* solid tumors and acute leukemia indicated a consistent safety profile and noted preliminary activity, particularly in acute myeloid leukemia (AML). This study also established recommended dosing regimens for subsequent combination studies [45]. HDM201 monotherapy in relapsed AML patients following allogeneic stem cell transplantation (allo-SCT) demonstrated consistent safety with no excessive graft vs. host disease (GvHD) and allowed continuation at full or reduced doses. Preliminary data suggest it has anti-leukemic activity, supporting further investigation as a potential maintenance or pre-emptive treatment to enhance graft-versus-leukemia effects [52]. Furthermore, a phase Ib proof-of-concept study investigating the co-targeting of MDM2 and CDK4/6 with HDM201 and ribociclib for patients with well-differentiated or dedifferentiated liposarcoma (WDLPS/DDLPS) demonstrated manageable toxicity and early signs of antitumor activity [49]. Current data and multiple ongoing clinical trials suggest that HDM201 is a promising candidate for the treatment of cancers with activatable functional p53. In this study, we assessed the effects of HDM201 on B cell lines and ex vivo patient-derived primary CLL cells and compared these effects with those observed in *TP53* mutant cells as well as on normal hematopoietic cells.

## 2. Materials and Methods

### 2.1. Cell Lines and Compound

Human B cell lines, including Nalm-6, OCI-Ly3, and HAL-01 (wild-type *TP53*), as well as Ramos, Raji, and Pfeiffer (*TP53* mutant), were acquired from authenticated cell line repositories (ATCC or DSMZ). Nalm-6 *TP53* monoallelic (+/−) and biallelic (−/−) isogenic knockout cell lines were purchased from Horizon Discovery (Cambridge, UK) and were generated using gene targeting by homologous recombination with an exon-2 deleted *TP53* DNA construct [53].

All these cell lines were cultured in RPMI-1640 medium (Sigma-Aldrich, St. Louis, MO, USA), supplemented with 10% fetal calf serum and 100 U/mL penicillin/streptomycin (Sigma-Aldrich, St. Louis, MO, USA). HDM201 (>99% purity) was obtained by a custom synthesis through the CRUK Drug Discovery Programme at the Newcastle University Cancer Centre and from Selleck Chem. The compound was accurately weighed and dissolved in dimethyl sulfoxide (DMSO; Sigma-Aldrich, St. Louis, MO, USA) to achieve a final concentration of 20 mM. From this stock solution, aliquots of lower concentrations were prepared and stored at −20 °C.

### 2.2. Cell Viability Assay for the Cell Lines

Cells were plated at a density of 0.2 × 10^6^ cells/mL in 100 μL of culture medium per well in a 96-well plate (Corning) and incubated for 24 h prior to treatment with HDM201 at concentrations ranging from 0 to 10 µM for a duration of 72 h at 37 °C. Growth inhibition was assessed using the XTT Assay Kit (Cayman Chemical Company, Ann Arbor, MI, USA) relative to a DMSO control. The optimal incubation time with the XTT reagents was determined to be 4 h for the cell lines through pre-optimization procedures. Data were normalized to the DMSO control and expressed as a percentage of relative growth. The mean ± standard error of the mean (SEM) for IC_50_ values from at least three independent experiments was calculated.

### 2.3. Patient Samples

Peripheral blood samples from patients with CLL were collected following informed consent and in accordance with institutional guidelines and the Declaration of Helsinki. This study was approved by the UK NHS Research Ethics Service, and research has been conducted using samples obtained through the Newcastle Biobank (Study ID: 17/NE/0361, Date of Approval: 29 April 2014). The diagnosis of CLL was confirmed based on the IWCLL-164 NCI 2008 criteria [54]. Peripheral blood samples were obtained from CLL patients with total white blood cell counts of at least 30 × 10^9^ cells/L, supporting the high proportion of malignant CD5+/CD19+ B cells. Mononuclear cells were purified by density gradient centrifugation (Lymphoprep, Axis-Shield Ltd., Dundee, UK) following the manufacturer’s protocol. Sensitivity to MDM2 inhibitors was always assessed on fresh samples just after collecting from the patients. The cells were cultured in RPMI-1640 medium (Sigma-Aldrich, St. Louis, MO, USA), supplemented with 10% fetal calf serum and 100 U/mL penicillin/streptomycin (Sigma-Aldrich, St. Louis, MO, USA). HDM201 was dissolved in DMSO (Sigma-Aldrich) and used at a final concentration of 0.5% (*v*/*v*) DMSO in the medium. This concentration of DMSO was previously demonstrated to have minimal cytotoxic effects on the cells [55].

Normal peripheral blood mononuclear cells (PBMCs) were isolated from three healthy donors. Normal PBMCs and CLL cells were isolated by density gradient centrifugation (Lymphoprep, Axis-Shield Ltd., Dundee, UK) following the manufacturer’s protocol.

### 2.4. Patient Sample Information

The details of Sanger sequencing of *SF3B1* in primary CLL samples have been described in a previous study [39]. The *TP53* mutational status of CLL samples was assessed by next-generation sequencing (using Roche 454 GS FLX and Illumina MiSeq platforms) in all samples. The functional status of p53 in CLL samples was determined by observing the stabilization of p53 and activation of downstream proteins, p21^WAF1^ and MDM2, following exposure to the MDM2 inhibitor HDM201. Viability was routinely assessed by trypan blue exclusion assay and was >95% in fresh and thawed samples after 24 h culture.

### 2.5. Cell Viability Assay for the Primary Cells

An amount of 5 × 10^6^ cells/mL in 100 μL of medium per well in a 96-well plate was exposed to a range of concentrations (from 0 to 3000 nM) of HDM201 at 37 °C for 48 h. Ex vivo cytotoxicity was assessed by the XTT Assay Kit (Cayman Chemical Company, Ann Arbor, MI, USA). The optimal incubation time with the XTT reagents was determined to be 8 h through pre-optimization procedures. Results were normalized to DMSO controls and expressed as % viability.

### 2.6. Immunoblotting

An amount of 5 × 10^6^ cells/mL primary CLL cells were seeded in 2 mL per well of a 6-well plate (Corning) and subjected to treatment with HDM201. Protein lysates were harvested using 2% SDS lysis buffer at 24 h, heated at 95 °C for 10 min, and sonicated. Protein concentration was measured using a Pierce™ BCA Protein Assay Kit (Thermo Fisher Scientific, Rugby, UK). Primary antibodies against p53 (DO-7) (#M7001, Dako, Santa Clara, CA, USA), MDM2 (Ab-1) (#OP46, Merck Millipore, Jaffrey, NH, USA), p21^WAF1^ (Calbiochem, San Diego, CA, USA), PARP (Trevigen, Gaithersburg, ML, USA), Actin (Sigma, Livonia, MI, USA), and secondary goat anti-mouse/rabbit horseradish peroxidase-conjugated antibodies (Dako, Santa Clara, CA, USA) were used. All antibodies were diluted in 5% (*w*/*v*) nonfat milk or BSA in TBS-tween20. Protein bands were visualized using enhanced chemiluminescence reagents (GE Healthcare, Alger, OH, USA).

### 2.7. Annexin V-FITC/PI Analysis

Peripheral blood mononuclear cells (PBMCs) from CLL patients were resuspended in pre-warmed medium and seeded at 5 × 10^6^ cells/mL in a 24-well plate. After a 1-h equilibration period, cells were treated with various drug concentrations for 48 h. Following treatment, cells were harvested, washed with cold PBS, and resuspended in 1× binding buffer (10 mM Hepes/NaOH (pH 7.4), 0.14 M NaCl, 2.5 mM CaCl_2_) at 1 × 10^6^ cells/mL. Cells (100 μL of 1 × 10^5^ cells) were transferred to round-bottom tubes and stained with 5 μL of annexin V-FITC (BD Pharmingen™, Hong Kong) and 5 μL of propidium iodide (PI, BD Pharmingen™, Hong Kong). Controls included unstained cells and cells stained with either annexin V-FITC or PI alone. After a 15-min incubation in the dark, 400 μL of 1× binding buffer was added for analysis within 1 h. Flow cytometry was conducted using an Attune™ NxT Acoustic Focusing Flow Cytometer. Data were acquired for 25,000 events per sample, with detection of annexin V-FITC on the FL1 channel (max emission wavelength = 519 nm; max excitation wavelength = 495 nm) and PI on the FL2 channel (max emission wavelength = 617 nm; max excitation wavelength = 536 nm). FCS files were analyzed with FCS Express 6 software.

### 2.8. Statistical Analysis

The data from the repeated experiments were presented as mean ± standard error of the mean (SEM) unless otherwise stated. Statistical tests were carried out using GraphPad Prism 6 software, and all *p*-values represent paired or unpaired *t*-tests of at least three independent repeats unless otherwise stated.

## 3. Results

### 3.1. TP53 Wild-Type B Cell Lines Demonstrate Sensitivity to MDM2 Inhibition Using HDM201

**Figure 1:**
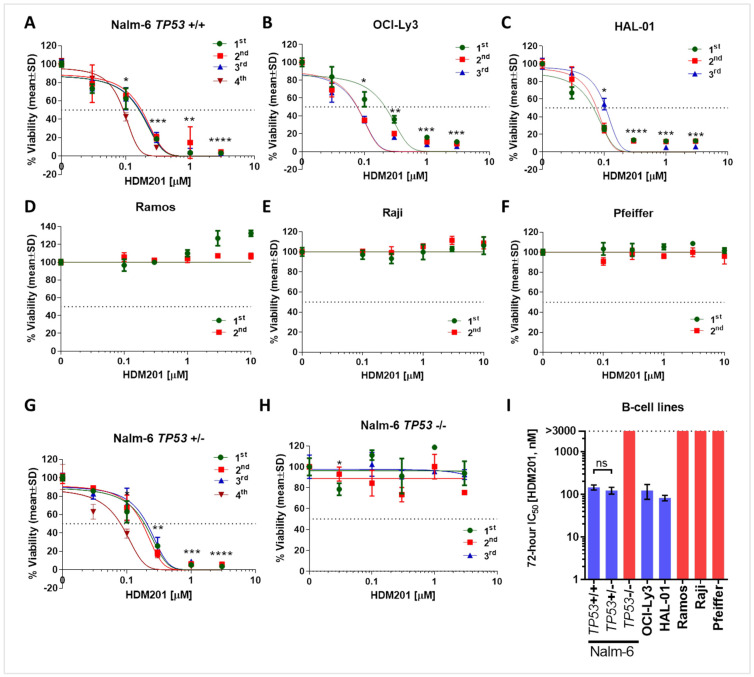
The concentration-dependent effect of HDM201 on the proliferation of a panel of B cell lines: (**A**) Nalm-6 *TP53 (*+/+), (**B**) OCI-Ly3, (**C**) HAL-01, (**D**) Ramos, (**E**) Raji, and (**F**) Pfeiffer. Isogenic NALM-6 cell lines with (**G**) *TP53* (+/−) and (**H**) *TP53* (−/−) genotypes were also incorporated into the growth inhibition assays. Each cell line shows independent repeats (e.g., 1st: green, 2nd: red, 3rd: blue, and 4th: brown). Bars show the mean IC_50_ ± SD. The asterisks show how significantly the drug induces cell death for each concentration compared to DMSO control. Each independent repeat of the experiment was averaged within itself, and then a paired *t*-test was applied to compare paired measurements (*, *p* ˂ 0.05; **, *p* ˂ 0.01; ***, *p* ˂ 0.001; ****, *p* ˂ 0.0001). (**I**) Summary IC50 values of HDM201 for the panel of B cell lines. The results for *TP53* wild-type and *TP53* mutant cell lines are shown in blue and red bars, respectively. Bars show the mean ± SEM. A standard parametric paired *t*-test was applied to compare *TP53*+/+ with *TP53*+/− Nalm-6 cells. ns, not significant.

Six different B cell lines were exposed to increasing concentrations of the MDM2 inhibitor HDM201. An XTT assay was used to measure the relative growth of the cells. Figure 1 displays growth inhibition curves for the cell lines treated with HDM201 for 72 h. Three out of the six B cell lines were sensitive to the MDM2 inhibitor, with IC_50_ values ≤ 146 nM (Figure 1A–C). In contrast, HDM201 had no significant effect on *TP53* mutant cell lines Ramos and Raji, nor on *TP53*-null Pfeiffer, even at concentrations up to 10 µM (Figure 1D–F). Figure 1I and Table 1 summarize the IC_50_ values of HDM201 and the *TP53* gene status for all cell lines used in this study.

To directly evaluate the effect of *TP53* status alone on the response of B cells to HDM201 while minimizing the impact of additional genomic variations, we utilized otherwise isogenic *TP53*-knockout (*TP53*-KO) derivatives of the human B cell line Nalm-6. These cells were treated with HDM201, and their responses were assessed and compared. Both *TP53*+/+ and heterozygous KO (*TP53*+/−) Nalm-6 cells exhibited sensitivity to the compound at low concentrations (mean ± SEM IC_50_s are 146 ± 20 nM and 123 ± 22 nM, respectively) (Figure 1I and Table 1; *TP53*+/+ vs. *TP53*+/− paired *t*-test *p* = 0.27). In contrast, homozygous KO (*TP53*−/−) cells showed markedly increased resistance to HDM201, with an IC_50_ exceeding 3 µM, the highest concentration tested (Figure 1H,I and Table 1).

### 3.2. TP53 Wild-Type Primary CLL Samples Are Sensitive to MDM2 Inhibition Using HDM201

**Figure 2:**
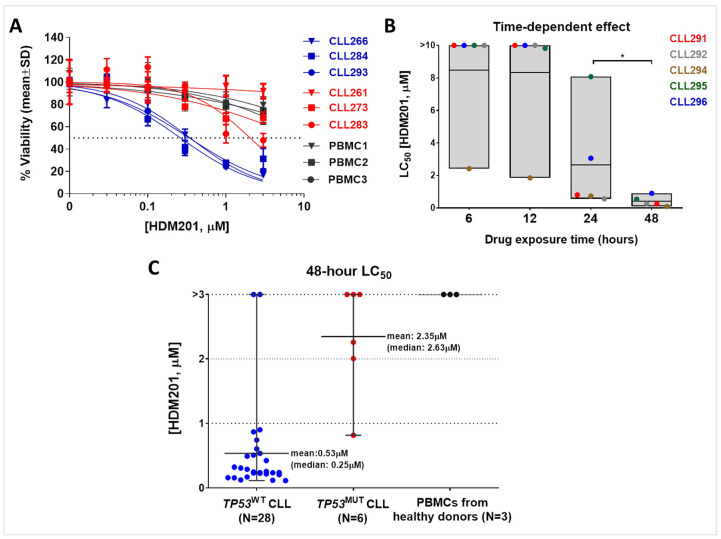
Time- and concentration-dependent effects of HDM201 on primary CLL samples: (**A**) Representative cytotoxicity curves for *TP53* wild-type (blue) and mutant (red) CLL samples. The samples were treated with increasing concentrations of HDM201 (ranging from 0 to 3 µM) for a duration of 48 h. To compare with the response of normal cells to HDM201, three PBMC samples (black) sourced from individual healthy donors were tested using the XTT assay. (**B**) LC50 values of HDM201 for each individual CLL sample across increasing exposure durations (6, 12, 24, and 48 h) are plotted. The time-dependent effects of HDM201 exposure were analyzed for the five CLL samples. Each CLL sample is a different color. A statistically significant difference in LC50 was observed between 24 h and 48 h exposure (paired *t*-test, denoted as * *p* < 0.05). (C) A summary of the LC50 values for the MDM2-p53 binding antagonist HDM201, tested on primary CLL samples with *TP53* wild-type (N = 28) and mutant (N = 6) profiles. The samples were subjected to increasing concentrations of HDM201 (ranging from 0 to 3 µM) for a 48-h exposure period. The three PBMC samples (indicated in black), derived from healthy donors, exhibited LC50 values exceeding 3 µM. Horizontal bars show mean values.

Ex vivo cytotoxicity of HDM201 was evaluated using an XTT assay after 48 h of treatment compared with untreated DMSO vehicle controls. HDM201 significantly and potently reduced the viability of *TP53* wild-type CLL cells but had minimal or no effect on cells with mutant *TP53* (Figure 2A,C). The LC_50_ values (concentration required for 50% loss of viability) for each primary CLL sample, as summarized in Figure 2C, indicate that *TP53* wild-type CLL cells overall were sensitive to HDM201-induced cytotoxicity (median LC_50_ = 0.253 µM), whereas CLL samples with mutated *TP53* were substantially more resistant (median LC_50_ = 2.63 µM; Figure 2C). Since the LC_50_ values did not follow a normal distribution (D’Agostino–Pearson omnibus test, *p* < 0.05), row median and mean values with the range bars are presented in Figure 2C. Detailed information on *TP53* mutations and LC_50_ values for each *TP53* mutant CLL sample is provided in Table 2. The LC_50_ data for all CLL primary samples utilized in this study are also provided in Supplementary Material Table S1.

To assess the effect of HDM201 on normal cells, PBMC samples from healthy donors were exposed to HDM201 for 48 h. In contrast to *TP53* wild-type CLL cells, normal PBMCs were resistant to HDM201 and exhibited LC_50_ values consistently greater than 3 μM (Figure 2A,C).

For evaluating the time-dependent effects of HDM201, five CLL patient samples with functional p53 were treated with various concentrations of HDM201 or left untreated. At different time points (6, 12, 24, or 48 h), the compound was washed out, and the cells were resuspended in complete medium without the drug. The cells were then plated in a 96-well format, and viability was assessed at 48 h using the XTT assay. These wash-out experiments showed that HDM201 decreased CLL viability in both a concentration-dependent and time-dependent manner (paired *t*-test, 24 h vs. 48 h, *p* = 0.02). Median LC_50_ values across CLL samples decreased over time, with LC_50_ values exceeding 10 μM for 6- and 12-h exposures (except for CLL294) but decreasing significantly with longer exposure periods (Figure 2B).

### 3.3. HDM201 Induces p53 Stabilization and Functional Activation in TP53 Wild-Type CLL Cells

**Figure 3:**
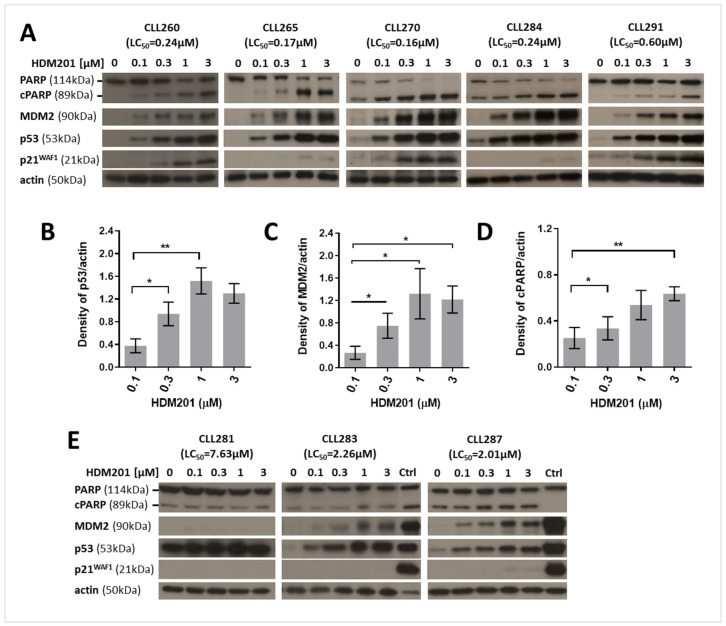
Western immunoblot analyses illustrating the response to HDM201 of both functional and non-functional p53 CLL samples: (**A**) Western immunoblots depicting *TP53* wild-type CLL cells, either untreated (DMSO control) or exposed to increasing concentrations of HDM201 (0.1, 0.3, 1, and 3 µM) for 24 h. Actin served as a loading control. Densitometry was performed for p53 (**B**), MDM2 (**C**), and cPARP (**D**) bands and normalized to actin. Statistical comparisons of different HDM201 concentrations were carried out using a paired *t*-test (*, *p* < 0.05; **, *p* < 0.01). Data are presented as mean ± SEM. (**E**) Representative Western immunoblots for *TP53* mutant CLL samples. In contrast to functional samples, there was no discernible increase in either the p21^WAF1^ or the cleaved PARP-1 (cPARP) marker of apoptotic signaling. Ctrl: Control lysate from OCI-Ly3 cells treated with 0.3 μM RG7388 (idasanutlin) for 24 h. Control lysates were run on the same gel with the sample shown beside the controls.

The functional integrity of the p53 signaling pathway was evaluated by treating CLL cells with the MDM2-p53 antagonist HDM201 for 24 h and assessing the protein expression of p53 transcriptional target genes. Inhibition of MDM2 led to the prevention of ubiquitin-mediated degradation of p53, resulting in p53 stabilization. In *TP53* wild-type CLL cells, HDM201 induced a concentration-dependent stabilization of p53, which was accompanied by the activation of p53 target genes, *CDKN1A* (encoding the p21^WAF1^ protein) and *MDM2* (Figure 3A). The concentration-dependent accumulation of p53 was consistently observed in all *TP53* wild-type CLL samples within 24 h of treatment (Figure 3A). To monitor apoptosis, PARP-1 cleavage was analyzed, revealing a concentration-dependent decrease in the levels of full-length PARP accompanied by an increase in the 85 kDa cleavage product (cPARP) in p53-functional CLL cells treated with HDM201 for 24 h. Densitometric analysis of p53, MDM2, and cPARP bands, normalized to actin, showed a significant concentration-dependent increase in these proteins when analyzed across five samples using paired *t*-tests (Figure 3B–D).

In contrast, CLL samples with *TP53* mutations exhibited no induction of p21^WAF1^ following treatment with HDM201, even at concentrations up to 3 μM (Figure 3E). The absence of p21^WAF1^ induction was noted in *TP53* mutant CLL cells from patients CLL281, CLL283, and CLL287. Additionally, there was no significant change in the levels of full-length PARP-1 or cPARP following 24 h of exposure of *TP53* mutant CLL cells to HDM201. Although some degree of p53 stabilization was observed in CLL283 and CLL287 samples after 24 h of HDM201 exposure, it did not result in downstream activation of p21^WAF1^. The markedly intense p53 band observed in sample CLL281, including the untreated control sample and the absence of both MDM2 and p21^WAF1^ signals, indicates the accumulation of mutant non-functional p53 (Figure 3E). It is important to note that the *TP53* mutant CLL samples analyzed include a mixed clonal population of *TP53* mutant and wild-type cells with varying variant allele frequencies (Table 2).

### 3.4. HDM201 Treatment Increases the Proportions of Early and Late Apoptotic CLL Cells

**Figure 4:**
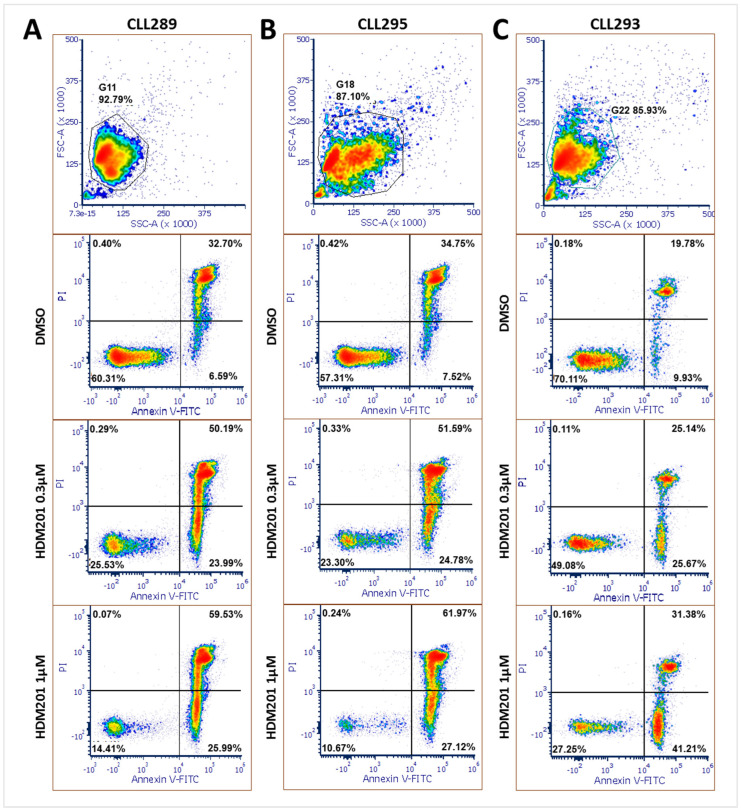
Induction of apoptosis in freshly isolated CLL samples CLL289 (**A**), CLL295 (**B**), and CLL293 (**C**) following treatment with HDM201. CLL cells were exposed to two distinct concentrations of HDM201 (0.3 µM and 1 µM) or a vehicle control (DMSO) for 48 h. Apoptosis was assessed by flow cytometry after staining with annexin V-FITC and PI. The lower left quadrants indicate viable cells (annexin V-negative/PI-negative), the lower right quadrants represent cells in the early stage of apoptosis (annexin V-positive/PI-negative), and the upper right quadrants denote cells in the late stage of apoptosis or necrosis (annexin V-positive/PI-positive). The percentages of the cell populations within each quadrant are presented.

Western blot analysis revealed a concentration-dependent increase in cPARP levels in p53-functional CLL cells following 24 h of HDM201 treatment. To further characterize the apoptotic effects of HDM201, flow cytometry was employed using annexin V and propidium iodide (PI) staining. The intensity of PI staining was plotted against annexin V staining to distinguish between viable (annexin V-negative/PI-negative), early apoptotic (annexin V-positive/PI-negative), and late apoptotic or necrotic (annexin V-positive/PI-positive) cells. Cell debris was excluded from the analysis, and the cell population of interest was identified based on forward and side scatter intensity (Figure 4). Figure 4A–C illustrate concentration-dependent changes in the proportion of cells staining positively for annexin V and/or PI, reflecting apoptosis induction in three freshly isolated CLL primary samples: CLL289, CLL295, and CLL293.

**Figure 5:**
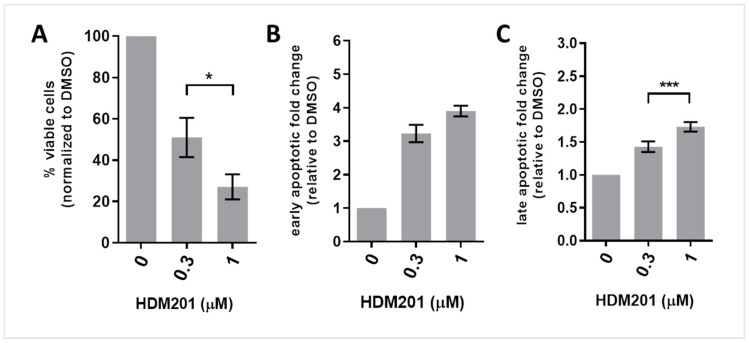
HDM201 induces apoptosis in CLL cells in a concentration-dependent manner. Freshly isolated CLL samples (*n* = 3) were treated with either 0.3 μM or 1 μM HDM201, or with a vehicle control (DMSO), for 48 h. Apoptosis was assessed using FACS analysis of annexin V/PI staining. (**A**) The percentage of viable cells remaining was calculated relative to untreated control samples. The fold change in the percentage of early (**B**) and late (**C**) apoptotic cells relative to the DMSO control is presented for the two different concentrations. Data are expressed as mean ± SEM. Statistically significant differences between concentrations were determined using a paired sample *t*-test (*, *p* < 0.05; ***, *p* < 0.001).

After 48 h of HDM201 treatment, a significant decrease in the proportion of viable cells was observed. The normalized percentages of viable cells decreased to 51 ± 10% (mean ± SEM) at 0.3 µM of HDM201 and to 27 ± 6% (mean ± SEM) at 1 µM of HDM201 (Figure 5A; 0.3 µM vs. 1 µM paired *t*-test, *p* = 0.02). Conversely, early apoptotic cell proportions (Figure 5B) and late apoptotic cell proportions (Figure 5C) relative to DMSO control increased following 48 h of HDM201 treatment.

A total of 73 ± 11% (mean ± SEM) of CLL cells lost viability at 1 μM HDM201 compared to the DMSO control (Figure 5A). The reduction in viability observed in these CLL samples, as measured by FACS analysis of annexin V/PI staining, showed a strong positive correlation with the results from the XTT cell viability assay (mean ± SD: 69 ± 6% viability decrease at 1 μM HDM201). This correlation was statistically significant (Pearson’s test: r = 0.99; *p* = 0.01; *n* = 3).

## 4. Discussion

The landscape of CLL treatment has undergone substantial evolution over the past decades, marked by a transition from traditional chemotherapy to more sophisticated targeted therapies. Despite significant advancements, challenges persist, particularly concerning resistance mechanisms and suboptimal responses in high-risk cases. Our study has focused on exploring the potential of HDM201, a second-generation MDM2-p53 binding antagonist, as a novel therapeutic strategy to include for CLL. The promise of HDM201 stems from its ability to specifically target the MDM2-p53 interaction, thereby stabilizing p53 and activating its tumor-suppressive functions in a non-genotoxic manner. This approach contrasts with traditional treatments that largely rely on direct cytotoxicity or modulation of immune responses. The rationale behind targeting the p53 pathway in CLL is grounded in the relatively low frequency of *TP53* alterations at diagnosis (approximately 10%), which suggests that a significant portion of CLL cases could benefit from therapies aimed at activating p53 function.

The safety profile of HDM201 observed in clinical trials appears promising, showing manageable toxicity and no severe adverse effects. This is crucial for CLL patients, who are often older and may have multiple comorbidities. The ability of HDM201 to be administered without excessive toxicity could make it a viable option for long-term or combination therapy, particularly in settings where current treatments have failed or have become less effective.

One of the initial aims of this study was to directly evaluate the effects of HDM201 on various B cell lines with differing *TP53* status, employing otherwise isogenic *TP53*-knockout (*TP53*-KO) derivatives of the human B cell line Nalm-6 to minimize the impact of other genomic variations. Our results demonstrated that both parental (*TP53* wild-type) and heterozygous KO (*TP53*+/−) Nalm-6 cells were sensitive to HDM201. In contrast, B cells with *TP53* mutations or deletions, including homozygous *TP53*-KO (*TP53*−/−) cells, showed a lack of response to HDM201. These findings underscore the crucial role of *TP53* in mediating the response to HDM201 and emphasize the specificity of the compound. Our results reinforce the notion that *TP53* status is a critical factor in determining the therapeutic efficacy of MDM2 inhibitors, highlighting the importance of assessing *TP53* status as a patient selection biomarker to predict the responsiveness of B cell malignancies to HDM201.

We also evaluated HDM201 in malignant B cells isolated from the peripheral blood of patients with CLL. Almost all *TP53* wild-type samples exhibited a response to the compound within clinically acceptable limits [45]. In contrast, *TP53* mutant samples demonstrated varying responses based on the specific mutation type and mutant allele frequency, with drug resistance being generally predominant. The clinical applicability of such treatments hinges on their selective toxicity toward malignant cells while sparing healthy cells. Previous studies have shown that MDM2 inhibitors effectively target malignant cells while sparing normal B cells and stem cells [38,56,57]. Although MDM2 inhibitors activate p53 in both normal and tumor cells with functional p53, the resulting gene expression and cytotoxic effects differ significantly between these cell types [38]. We have previously shown that MDM2 inhibition selectively induces a pro-apoptotic p53 gene signature in CLL cells [38]. This difference leads to distinct cell fates and provides a therapeutic window that has important implications for the use of MDM2 inhibitors as potential cancer treatments. This mechanism is supported by the reversible growth arrest in normal cells as the primary response to MDM2 inhibition, observed with both first-generation and second-generation MDM2 antagonists [38,43,58]. To further investigate this, we isolated peripheral B cells from three different healthy donors and treated them with HDM201 to assess its effects on normal healthy B cells. Remarkably, all isolated B cells displayed resistance to the drug. These findings are significant as they underline the selective toxicity and clinical relevance of targeted therapies, supporting prior research on MDM2 inhibitors.

One notable challenge revealed by our preclinical study is the potential for resistance mechanisms, a common issue across various targeted therapies. In our analysis of 28 primary CLL samples with wild-type *TP53*, we identified two samples exhibiting notable resistance to HDM201 (Figure 2C). Previous research has demonstrated that *SF3B1* mutations, alongside *TP53* status, are an important determinant of response to MDM2 inhibitors [59]. To further explore this, we checked the *SF3B1* gene status of these two resistant samples from our previous comprehensive sequencing data. Notably, one of these two samples, CLL290, was found to carry the *SF3B1* c.2110A > T (p.I704F) point mutation [59]. Despite being *TP53* wild-type, this sample showed significant resistance not only to HDM201 but also to another MDM2 inhibitor, RG7388 (idasanutlin), with an LC_50_ exceeding 10 µM [59]. These results further emphasize the substantial impact of *SF3B1* mutations on the effectiveness of MDM2 inhibitors and underscore the importance of identifying and considering additional biomarkers of treatment response.

The role of HDM201 as an MDM2-p53 binding antagonist is to stabilize p53 in *TP53* wild-type CLL cells by preventing its ubiquitin-mediated degradation. This stabilization was demonstrated in our study and promoted the increased expression of key downstream TP53 transcriptional targets CDKN1A (p21^WAF1^) essential for inhibition of cell cycle progression and the futile increased expression of the negative feedback autoregulator MDM2. PARP-1 is one of several known cellular substrates of caspases. Cleavage of PARP-1 by caspases is considered to be a hallmark of apoptosis [60]. As a marker of caspase-dependent apoptosis, we evaluated PARP-1 cleavage in Western blot experiments. The increased levels of cPARP in *TP53* wild-type CLL cells provided mechanistic evidence of the pro-apoptotic effect of HDM201. These findings were corroborated by flow cytometry, Western blot, and XTT assay results, demonstrating the capacity of HDM201 to induce apoptosis through the activation of the p53 pathway. Conversely, *TP53* mutant CLL cells exhibited resistance to HDM201, consistent with the lack of p21^WAF1^ induction and minimal apoptosis (Figure 3). *TP53* mutations result in dysfunctional p53, impairing its ability to trigger downstream apoptotic pathways. The intense p53 bands in our *TP53* mutant samples, especially in sample CLL281, indicate the accumulation of non-functional and conformationally altered p53, which is no longer recognized by MDM2 and, therefore, not able to be targeted for normal turnover degradation. This is consistent with the known behavior of some mutant forms of TP53 and associated resistance mechanisms. The clonal heterogeneity observed in *TP53* mutant CLL samples (Table 2) complicates the overall effect of treatment, as subclones will differ in their response to HDM201. This complexity underlines the need for tailored therapeutic strategies to address clonal resistance and optimize treatment efficacy by choosing appropriate targeted drug combinations in genetically diverse CLL populations.

In a recently published study, we conducted a detailed transcriptome analysis of a small subpopulation of CLL samples resistant to the MDM2 inhibitor RG7388 and identified several candidate genes [61]. Although these genes are also potential candidates for HDM201, which operates through the same mechanism as RG7388, further research is needed to thoroughly characterize their roles and validate their relevance to the efficacy of HDM201. Further evaluation of apoptotic and DNA damage response pathways could be highly beneficial, particularly for elucidating resistance mechanisms in the limited number of samples and for a more mechanistic understanding of drug response. Future research should focus on delineating the precise mechanisms through which resistance to HDM201 and other MDM2 inhibitors may develop and seek to identify biomarkers that predict response or resistance. This could involve evaluating the impact of co-occurring mutations or the role of the microenvironment in influencing drug efficacy. Furthermore, combination strategies involving HDM201 and other targeted agents, such as BTK inhibitors or novel immunotherapies, could potentially enhance therapeutic outcomes and overcome resistance barriers.

## 5. Conclusions

Here, we present a rationale for the further evaluation of the new-generation MDM2 inhibitor HDM201 in CLL therapy. Our observations indicate that CLL cells are particularly primed for p53-dependent apoptosis compared to normal PBMCs. Given the promising results observed in B cell lines and primary patient samples, HDM201 could become a valuable addition to the therapeutic arsenal for CLL. Integrating HDM201 into treatment regimens may offer a new strategy for managing relapsed or refractory cases. These results support the investigation of MDM2 inhibitors in CLL clinical trials, which will determine their optimal use in clinical treatment strategies.

## Figures and Tables

**Table 1:** Mean IC_50_ concentrations of HDM201 for the panel of B cell lines.

Cell Line	*TP53* Gene Status	HDM201 IC_50_ (nM)
OCI-Ly3	Wild-type	123 ± 47
HAL-01	Wild-type	83 ± 12
Nalm-6	+/+	146 ± 20
	+/−	123 ± 22
	−/−	>3000
Ramos	Mutant (Homozygous) c.761T > A; p.I254N	>10,000
Raji	Mutant (Heterozygous) c.638G > A; p.R213Q c.700T > C; p.Y234H	>10,000
Pfeiffer	Null c.(del)	>10,000

The IC_50_ values shown represent the mean of at least *n* = 3 independent repeats ± SEM.

**Table 2:** Detailed information on *TP53* mutations and LC_50_ values for each *TP53* mutant CLL sample.

Tumor ID	^1^ VAF (%)	^2^ CDS Mutation	Amino Acid Mutation	HDM201 LC_50_ (µM)
CLL258	35	c.626_627delGA	p.R209fs*6 (Deletion—Frameshift)	0.82
CLL261	97	c.626_627delGA	p.R209fs*6 (Deletion—Frameshift)	>3
CLL273	20	c.1067G > C	p.G356A	>3
	20	c.1069A > C	p.K357Q	
CLL281	28	c.623A > T	p.D208V	>3
	66	c.659A > G	p.Y220C	
CLL283	48	c.745A > G	p.R249G	2.26
CLL287	50	c.524G > A	p.R175H	2.01

^1^ VAF: variant allele frequency; ^2^ CDS: coding sequence.

## Data Availability

The data presented in this study and released under a CC-BY 4.0 license are available upon request from the corresponding authors.

## Supplementary Materials

The following supporting information can be downloaded at https://www.mdpi.com/article/10.3390/cancers17020274/s1: Figure S1: Densitometry readings; Table S1: Detailed information on *TP53* mutations and LC_50_ values for each CLL sample.
