# Supplementary material for: Targeting the MDM2-p53 Interaction with Siremadlin: A Promising Therapeutic Strategy for Treating TP53 Wild-Type Chronic Lymphocytic Leukemia
Source: Cancers (Basel). 2025 Jan 16;17(2):274. doi: 10.3390/cancers17020274 (PMC11763703; doi:10.3390/cancers17020274)
Supplement: Supplementary file 1 [file cancers-17-00274-s001.zip › Figure S1.pdf]

p53	CLL260	CLL265	CLL270	CLL284	CLL291		CLL260	CLL265	CLL270	CLL284	CLL291
**0.1µM**	1225	1461	2987	5339	3654	**p53/actin**	*0,1458*	*0,2221*	*0,3732*	*0,8330*	*0,2806*
**0.3µM**	2954	5643	9081	7710	7398		*0,3293*	*0,9333*	*1,1491*	*1,3574*	*0,5689*
**1µM**	5619	13742	17464	15128	10013		*0,8129*	*2,0000*	*2,2982*	*2,1855*	*0,6886*
**3µM**	8398	11878	15961	13659	13969		*1,0146*	*1,5388*	*2,0014*	*1,7038*	*0,9333*

MDM2											
**0.1µM**	844	1009	2505	4044	1001	**MDM2/actin**	*0,1005*	*0,1534*	*0,3130*	*0,6310*	*0,0769*
**0.3µM**	2694	4039	8786	8999	3782		*0,3003*	*0,6680*	*1,1117*	*1,5843*	*0,2908*
**1µM**	2126	7871	20540	16844	5781		*0,3076*	*1,1455*	*2,7030*	*2,4334*	*0,3976*
**3µM**	3267	9649	17951	14613	7527		*0,3947*	*1,2500*	*2,2509*	*1,8228*	*0,5029*

cPARP											
**0.1µM**	747	828	3742	2171	806	**cPARP/actin**	*0,0889*	*0,1259*	*0,4675*	*0,3387*	*0,0619*
**0.3µM**	1611	1231	5039	2988	1376		*0,1796*	*0,2036*	*0,6376*	*0,5261*	*0,1058*
**1µM**	2557	6804	5726	4977	1102		*0,3699*	*0,9902*	*0,7535*	*0,7190*	*0,0758*
**3µM**	5012	5718	6143	4823	5174		*0,6055*	*0,7408*	*0,7703*	*0,6016*	*0,3457*

actin					
**0.1µM**	8401	6579	8004	6409	13023
**0.3µM**	8970	6046	7903	5680	13005
**1µM**	6912	6871	7599	6922	14541
**3µM**	8277	7719	7975	8017	14968
