# Supplementary material for: Targeting the MDM2-p53 Interaction with Siremadlin: A Promising Therapeutic Strategy for Treating TP53 Wild-Type Chronic Lymphocytic Leukemia
Source: Cancers (Basel). 2025 Jan 16;17(2):274. doi: 10.3390/cancers17020274 (PMC11763703; doi:10.3390/cancers17020274)
Supplement: Supplementary file 1 [file cancers-17-00274-s001.zip › Table S1.pdf]

Table S1. Detailed information on *TP53* mutations and LC_{50} values for each CLL sample.

Tumour ID	[1]VAF (%)	[2]CDS mutation	Amino acid mutation	HDM201 LC_{50} (μM)
CLL258	35	c.626_627delGA	p.R209fs*6 (Deletion-Frameshift)	0.82
CLL259				0.31
CLL260				0.24
CLL261	97	c.626_627delGA	p.R209fs*6 (Deletion-Frameshift)	**>3**
CLL264				0.51
CLL265				0.17
CLL266				0.12
CLL267				0.23
CLL268				0.12
CLL269				0.74
CLL270				0.16
CLL272				0.25
CLL273	20	c.1067G>C	p.G356A	**>3**
	20	c.1069A>C	p.K357Q	
CLL276				0.49
CLL277				>3
CLL281	28	c.623A>T	p.D208V	**>3**
	66	c.659A>G	p.Y220C	
CLL282				0.32
CLL283	48	c.745A>G	p.R249G	**2.26**
CLL284				0.24
CLL285				0.29
CLL286				0.16
CLL287	50	c.524G>A	p.R175H	**2.01**
CLL289				0.42
CLL290				>3
CLL291				0.60
CLL292				0.26
CLL293				0.24
CLL294				0.11
CLL295				0.53

CLL296				0.90
CLL298				0.87
CLL301				0.22
CLL302				0.21
CLL303				0.23

[1]VAF: variant allele frequency. [2]CDS: coding sequence.
